# Predictors of Poor Sleep Quality in Heart Failure Patients: A Cross-sectional Multivariable Analysis of Clinical, Demographic, and Psychosocial Factors

**DOI:** 10.34172/jrhs.2025.184

**Published:** 2025-04-01

**Authors:** Soroush Najdaghi, Delaram Narimani Davani, Davood Shafie

**Affiliations:** ^1^Heart Failure Research Center, Cardiovascular Research Institute, Isfahan University of Medical Science, Isfahan, Iran

**Keywords:** Heart failure, Sleep quality, Sleep disorders, Anxiety, Depression

## Abstract

**Background:** Sleep quality is crucial in heart failure (HF) patients, yet its associations with clinical, demographic, and psychosocial factors remain underexplored. This study examined these relationships to identify predictors of poor sleep quality.

**Study Design:** A cross-sectional study.

**Methods:** This cross-sectional was conducted on 354 HF patients at Shahid Chamran Heart Hospital, Isfahan, Iran (September 2023-2024). Pittsburgh Sleep Quality Index (PSQI) and Hospital Anxiety and Depression Scale (HADS) were used to evaluate sleep quality, anxiety, and depression, respectively. Random forest (RF) modeling and ordinary least squares (OLS) regression identified predictors of poor sleep quality.

**Results:** Poor sleep quality (PSQI>7) was observed in 18% of patients who were older (70.00±6.30, *P*<0.001) and had lower ejection fraction (EF) (23.75±12.79%, *P*<0.001). This group also had higher systolic blood pressure (BP 140.67±12.50 mmHg, *P*=0.014). Complex medication regimens, including angiotensin-converting enzyme (ACE) inhibitors, beta-blockers, and diuretics, were associated with poor sleep (*P*<0.001). Moreover, depression (HADS-depression: 5.36±0.70, *P*<0.001) and anxiety (HADS-anxiety: 4.84±1.32, *P*<0.001) were correlated with poor sleep. The RF model had an area under the curve of 0.79, and OLS regression (R^2^=0.280) highlighted New York Heart Association (NYHA) class and medication type as significant predictors.

**Conclusion:** Overall, poor sleep quality in HF patients was related to older age, reduced cardiac function, higher blood pressure (BP), complex medication regimens, and increased anxiety and depression. Accordingly, multidimensional management strategies are needed to improve sleep outcomes.

## Background

 Heart failure (HF) remains a significant public health issue, affecting over 64 million people globally in 2022, with an expected increase to nearly 5.7 million cases by 2030.^1,2^ It is often accompanied by psychological distress, with around 10%–70% of patients experiencing sleep disturbances such as insomnia and breathing difficulties.^1^ A recent umbrella review has demonstrated that sleep issues are linked to poorer quality of life, increased depression and anxiety, and a higher risk of myocardial infarction, highlighting its importance in multiple health outcomes.^3^

 Poor sleep quality in HF patients is associated with a 2.5-fold increase in hospitalization rates and a 1.7-fold increase in mortality.^4^ The causes of sleep disturbances are multifactorial, involving physiological factors such as nocturnal dyspnea and fluid retention and psychological stressors inherent in chronic disease management.^5^ In addition, depression and anxiety are prevalent in HF patients, with ranges of 20%–45% and 20%–50% for depression and anxiety, respectively.^6^ These mental health conditions are linked to poorer treatment adherence, reduced quality of life, and worse HF outcomes, creating a feedback loop where sleep disturbances exacerbate psychological distress, further deteriorating sleep quality and contributing to HF progression.^7,8^

 Screening for sleep issues is essential for developing effective management strategies.^9^ Moreover, psychological distress mediates the relationship between symptom burden and sleep quality, suggesting that addressing mental health could improve sleep outcomes.^10^ In the hospitalized setting, studies have found that poor sleep is widespread among HF patients, impacting up to 96%, though it often improves post-discharge.^11^ Additionally, a combination of conflicting findings in clinical, psychological, and demographic factors in hospitalized patients signifies their complexity across populations with different methodologies. For instance, while studies imply poor quality of sleep in older-aged, female gender, and hypertension (HTN) patients,^12-14^ others indicate no significant correlation between age, gender, and blood pressure (BP).^15-17^ Given this complexity, interventions such as motivational interviewing have shown mixed results in improving sleep quality and mental health in HF patients,^18^ highlighting the importance of tailored strategies to improve outcomes and reduce rehospitalization rates.^19^ In contrast, some studies argue that interventions targeting sleep quality may not always yield significant improvements in survival rates, underlining the multifaceted nature of sleep disorders in this population.^20^

 Predicting poor sleep quality in HF patients can also be effectively approached using machine learning and statistical models. These methodologies leverage various physiological indicators, such as electrocardiograms (ECGs) and actigraphic data, to forecast sleep quality, which is crucial for managing HF symptoms and improving patient outcomes. Support vector machines (SVMs) and polynomial regression have analyzed ECG signals to predict sleep quality, achieving significant accuracy in forecasting sleep stages and quality.^21^ Machine-learning models utilizing actigraph data (e.g., sleep duration and awakenings) have demonstrated an accuracy of 80%–86% in classifying sleep quality, which is vital for HF patients experiencing frequent sleep disturbances.^22^

 Given these findings and the necessity to conduct a larger observational study, this study aims to assess the clinical predictors and prevalence of poor sleep quality and its association with depression and anxiety in hospitalized HF patients. Further, the machine-learning model random forest (RF) has been utilized to identify important features predicting sleep disturbances in HF patients. Understanding these relationships will inform targeted interventions to improve psychological well-being and clinical outcomes, ultimately establishing a standard for managing sleep disturbances in this vulnerable population.

## Methods

 This study complies with the strengthening of the reporting of observational studies in epidemiology^23^.

###  Study design and setting

 This descriptive cross-sectional study was performed at Chamran Heart Hospital, Isfahan, Iran, from September 2023 to September 2024, involving 354 patients admitted to the HF ward under the care of an expert cardiologist for detailed evaluation. The sampling method was based on the use of questionnaires adapted and validated for the Iranian population, including the Hospital Anxiety and Depression Scale (HADS) and Pittsburgh Sleep Quality Index (PSQI).

 The study included all patients diagnosed with HF by a specialist and admitted to the ward during the study period. The inclusion criteria were documented HF diagnosis in medical records, history of HF for at least six months prior to admission, age ≥ 18 years, and no history of thyroid disorders, severe depression, schizophrenia, or neuropsychiatric disorders. The other criteria included no history of drug abuse, the ability to communicate and complete a written questionnaire, and no conditions affecting sleep quality (e.g., common cold, allergic rhinitis, recent psychological trauma).

 The exclusion criteria were medical records missing ≥ 10% of required data, cognitive impairments, dementia, delirium, or similar conditions, cerebrovascular or central nervous system diseases, malignant tumors, patients in intensive care or with unstable conditions, severe mental disorders (depression, schizophrenia, and neuroses), refusal or withdrawal of consent, newly diagnosed HF or HF diagnosis < 6 months.

###  Data collection and measurement

 The required data were meticulously gathered through a combination of medical record reviews and direct patient interviews. The collected demographic and clinical variables provided a comprehensive profile of each participant and included age, gender, ejection fraction (EF), New York Heart Association (NYHA) classification, medication type, HTN, diabetes, chronic kidney disease (CKD), smoking status, hemoglobin A1C, troponin, and C-reactive protein (CRP). Other clinical parameters were N-terminal prohormone of brain natriuretic peptide (NT-proBNP), estimated glomerular filtration rate (eGFR), hospitalization time, body mass index (BMI), education level, marital status, family history of early coronary heart disease (CHD), and first-day systolic and diastolic BP (SBP/DBP). The presence of CHD in immediate family members before the age of 55 will be recorded, providing insight into genetic predispositions.

 The HADS comprises 14 items divided into anxiety and depression subscales. Each item is rated on a 4-point scale, with a maximum score of 21 for each subscale. Scores ≥ 11 indicate significant psychological morbidity, 8–10 are borderline, and 0–7 are normal. The Iranian version of HADS is well-accepted by patients, with Cronbach’s alpha coefficients of 0.78 and 0.86 for anxiety and depression, respectively.^24^

 Sleep quality was measured using the PSQI, a standardized questionnaire with seven components assessing sleep difficulties. The PSQI global score ranges from 0 to 21. Overall, the Cronbach’s alpha for the Persian version of PSQI was 0.77, with 0.52 and 0.78 for patients and controls, respectively, indicating acceptable reliability.^25^

###  Statistical methods

 Descriptive statistics were used to summarize the clinical and demographic characteristics of the study population. Continuous variables were reported as means ± standard deviations (SD), and categorical variables were expressed as frequencies and percentages. To compare characteristics between patients with poor (PSQI > 7) and good (PSQI ≤ 7) sleep quality, independent t-tests were utilized for continuous variables, and Chi-square tests were employed for categorical variables.

 Comparisons of HADS subscores for anxiety and depression between patients with and without poor sleep quality were also conducted using independent t-tests. Forest plots were generated to visualize effect sizes and confidence intervals for associations with poor sleep quality.

 Multivariable logistic regression models were constructed to examine the relationship between poor sleep quality (the dependent variable) and various predictors across five models. These models adjusted for a range of variables, including:

- Model 1: Adjusted for age and gender
- Model 2: Model 1 + overweight or obesity (BMI ≥ 24 kg/m^2^), education, marital status, hospitalization time, and family history of early-onset CHD
- Model 3: Model 2 + smoking status, HTN, diabetes, CKD, CRP, and left ventricular EF
- Model 4: Model 3 + HADS-depression
- Model 5: Model 3 + HADS-anxiety

Ordinary least squares (OLS) regression analysis was performed to investigate associations between clinical, demographic, and treatment variables with poor sleep quality. Partial dependence plots (PDPs) were used to visualize the marginal effects of predictor variables on poor sleep quality, and interaction analyses were conducted to assess the interaction effect of anxiety and depression on sleep quality.

 An RF model was constructed to predict poor sleep quality. Model performance was evaluated using the receiver operating characteristic (ROC) curve and the area under the curve (AUC) to assess discriminative ability. A confusion matrix was utilized to calculate classification metrics, including precision, recall, F1-score, and overall accuracy.

 All statistical analyses were performed using Python (version 3.12), with packages such as ‘pandas’, ‘scipy’, ‘seaborn’, and ‘statsmodels’. A *P-*value of less than 0.05 was considered statistically significant.

## Results

###  Demographic and clinical characteristics

 Our study comprised 354 hospitalized HF patients with a mean age of 63.08 ± 7.55 years. Patients with poor sleep quality (PSQI > 7) were significantly older, with a mean age of 70.00 ± 6.30 years, compared to 62.58 ± 7.39 years in those with good sleep quality (*P <*0.001). This significant age difference underscores age as a key determinant of sleep quality in this patient population (Table 1).

**Table 1:** Demographic Characteristics of Hospitalized Patients with Heart Failure

**Variables**	**Total (N=354)**		**PSQI>7 (n=64)**		**PSQI≤7 (n=290)**		***P*** ** value**
**Continuous variables**	**Mean**	**SD**	**Mean**	**SD**	**Mean**	**SD**	
Age (year)	63.08	7.55	70.00	6.30	62.58	7.39	0.000
Ejection fraction	39.60	16.55	23.75	12.79	40.76	16.21	0.000
HADS subscore							
Anxiety	4.34	0.75	4.84	1.32	4.30	0.68	0.000
Depression	5.12	0.43	5.36	0.70	5.10	0.40	0.004
HbA1C	5.88	0.76	5.92	0.78	5.88	0.76	0.774
Troponin	0.64	0.48	1.07	0.41	0.61	0.47	0.000
CRP	5.92	2.02	6.56	2.46	5.88	1.98	0.108
NT-proBNP	796.35	388.58	1062.61	401.90	776.99	381.01	0.000
eGFR	81.93	8.23	82.31	8.49	81.90	8.22	0.814
Hospitalization time	15.18	5.60	19.72	5.28	14.85	5.49	0.000
BMI (k/m^2^)	25.95	3.07	25.38	2.56	25.99	3.10	0.342
First_Day_SBP	134.38	13.07	140.67	12.50	133.92	13.01	0.014
First_Day_DBP	88.78	7.33	91.52	6.60	88.58	7.35	0.057
Categorical variables	**Number**	**%**	**Number**	**%**	**Number**	**%**	***P *** **value**
Gender							
Male	185	52.26	32	50.00	153	52.73	0.659
Female	169	47.74	32	50.00	137	47.27	0.659
EF classification							
Mild	180	50.85	25	39.06	155	53.33	0.000
Moderate	68	19.21	11	17.19	57	19.70	0.000
Severe	106	29.94	28	43.75	78	26.97	0.000
NYHA class							
I	156	44.07	21	32.81	135	46.67	0.000
II	137	38.70	23	35.94	114	39.39	0.000
III/IV	61	17.23	21	32.81	40	13.94	0.000
Education							
University and above	182	51.41	33	51.56	149	51.52	0.141
Junior high school and below	133	37.57	27	42.19	106	36.67	0.141
High school/secondary school	39	11.02	5	7.81	34	11.82	0.141
Marital status							
Married	292	82.49	55	85.94	237	81.82	0.343
Single	62	17.51	9	14.06	53	18.18	0.343
Family history of early CHD							
No	221	62.43	41	64.06	180	62.12	0.821
Yes	133	37.57	23	35.94	110	37.88	0.821
Medication type							
ACE inhibitors/ARBs	111	31.36	15	23.44	96	33.03	0.000
ACE inhibitors/ARBs, and SGLT2 inhibitors	104	29.38	14	21.87	90	30.91	0.000
ACE inhibitors/ARBs, beta-blockers, diuretics, MRAs, ARNI, and SGLT2 inhibitors	36	10.17	12	18.75	24	8.18	0.000
ACE inhibitors/ARBs, beta-blockers, diuretics, MRAs, and ARNI	25	7.06	8	12.5	17	5.76	0.000
ACE inhibitors/ARBs, beta-blockers, MRAs, and SGLT2 inhibitors	25	7.06	5	7.81	20	6.97	0.000
ACE inhibitors/ARBs, beta-blockers, and MRAs	20	5.65	2	3.12	18	6.06	0.000
ACE inhibitors/ARBs, beta-blockers, and SGLT2 inhibitors	20	5.65	3	4.69	17	5.76	0.000
ACE inhibitors/ARBs, and beta-blockers	13	3.67	3	4.69	10	3.33	0.000

*Note*. ACE: Angiotensin-converting enzyme; ARBs: Angiotensin II receptor blockers; ARNI: Angiotensin receptor-neprilysin inhibitors; CHD: Coronary heart disease; DBP: Diastolic blood pressure; eGFR: Estimated glomerular filtration rate; HADS: Hospital anxiety and depression scale; MRAs: Mineralocorticoid receptor antagonists; NT-proBNP: N-terminal pro b-type natriuretic peptide; NYHA: New York Heart Association; PSQI: Pittsburgh sleep quality index; SBP: Systolic blood pressure; SGLT2: Sodium-glucose cotransporter 2;HbA1C: Hemoglobin A1C; SD: Standard deviation; EF: Ejection fraction; CRP: C-reactive protein; BMI: Body mass index.

###  Gender distribution

 The gender distribution in this cross-sectional study was 52.26% males and 47.74% females. Among patients with poor sleep quality, 54.17% were female and 45.83% were male. However, this difference was not statistically significant (*P*= 0.659), suggesting that gender does not significantly influence sleep quality in HF patients (Table 1).

###  Cardiac function: ejection fraction

 The mean EF across all patients was 39.60 ± 16.55%. Notably, patients with poor sleep quality exhibited a significantly lower mean EF of 23.75 ± 12.79%, compared to 40.76 ± 16.21% in those with good sleep quality (*P* < 0.001). The EF classification revealed that 50.85%, 29.94%, and 19.21% of patients had mild, severe, and moderate HF, respectively. The higher prevalence of severe HF among patients with poor sleep quality highlights a strong correlation between reduced cardiac function and impaired sleep quality (Table 1).

###  Blood pressure: First-day systolic and diastolic blood pressure

 The analysis of the admission (first-day) BP demonstrated that the mean SBP was significantly higher in patients with poor sleep quality (140.67 ± 12.50 mm Hg) than in those with good sleep quality (133.92 ± 13.01 mm Hg, *P* = 0.014). Although the mean DBP was also higher in the poor sleep quality group (91.52 ± 6.60 mm Hg) compared to the good sleep quality group (88.58 ± 7.35 mm Hg), this difference approached but did not reach statistical significance (*P* = 0.057, Table 1).

###  Medication use and sleep quality

 Medication analysis showed varying impacts on sleep quality. For instance, 31.36% of patients were on angiotensin-converting enzyme (ACE) inhibitors/angiotensin receptor blockers (ARBs) alone, while others were on combinations with sodium-glucose co-transporter 2 (SGLT2) inhibitors, beta-blockers, or diuretics. Notably, a combination of ACE inhibitors/ARBs with beta-blockers and diuretics was more common in patients with poor sleep quality (37.50%) compared to those with good sleep quality (8.18%, *P* < 0.001), suggesting that complex medication regimens may be associated with poorer sleep quality, possibly due to side effects or the severity of the underlying condition (Table 1).

###  Hospitalization duration and sleep quality

 Patients with poor sleep quality experienced longer hospitalization durations, with a mean of 9.12 ± 3.98 days compared to 8.13 ± 3.52 days for those with good sleep quality (*P* = 0.048). This extended hospitalization duration may reflect more severe disease states or complications contributing to poor sleep (Table 1).

###  Comorbidities and sleep quality

 HTN was present in 51.98% of the total patients, with a higher prevalence in the poor sleep quality group (66.67%) than in the good sleep quality group (50.91%). However, this difference was not statistically significant (*P* = 0.200). Similarly, diabetes prevalence did not differ significantly between the poor (58.33%) and good (51.82%) sleep quality groups (*P* = 0.685). However, CKD was more common in patients with poor sleep quality (70.83%) than in those with good sleep quality (50.00%), indicating a potential association between renal dysfunction and sleep disturbances (*P* = 0.078, Table 1).

###  Psychosocial factors: Depression and anxiety

 Depression and anxiety were strongly associated with poor sleep quality (*P* < 0.001). Patients with poor sleep quality had significantly higher depression scores (HADS-depression: 5.36 ± 0.70) compared to those with good sleep quality (5.10 ± 0.40). Anxiety scores were also higher in the poor sleep quality group (HADS-anxiety: 4.84 ± 1.32) versus the good sleep quality group (4.30 ± 0.68, *P* < 0.001). Moreover, poor sleep frequency was observed in 18% of the included patients. These findings highlight the critical role of psychosocial factors in sleep disturbances among HF patients (Table 1).

###  Functional status: New York Heart Association classification

 A significant association was observed between the NYHA classification and sleep quality. Patients with NYHA class III-IV were more likely to report poor sleep quality (62.50%) compared to those with NYHA class I-II (37.5%), suggesting that worsening HF symptoms were correlated with poorer sleep quality (*P* < 0.001, Table 1).

###  Correlation of psychosocial and clinical variables

 Pearson correlation analysis revealed low-to-moderate correlations between HADS-anxiety, HADS-depression, and various clinical and demographic variables. The correlation between HADS-anxiety and HADS-depression was low (r = -0.015), representing these conditions may manifest independently. Both HADS scores showed negligible correlations with cardiac biomarkers [HADS-depression with troponin (r = 0.043) and NT-proBNP (r = 0.045), and HADS-anxiety with troponin (r = 0.028) and NT-proBNP (r = -0.078)]. Correlations with CRP (r = -0.012) and eGFR (r = 0.035) were also weak, indicating minimal associations with inflammation and renal function. Age had a slight influence on HADS scores (r = -0.0036 for HADS-anxiety, r = 0.066 for HADS-depression). Hospitalization time demonstrated moderate positive correlations with age (r = 0.64) and NT-proBNP (r = 0.73).

###  Performance of the random forest model

**Figure 1:**
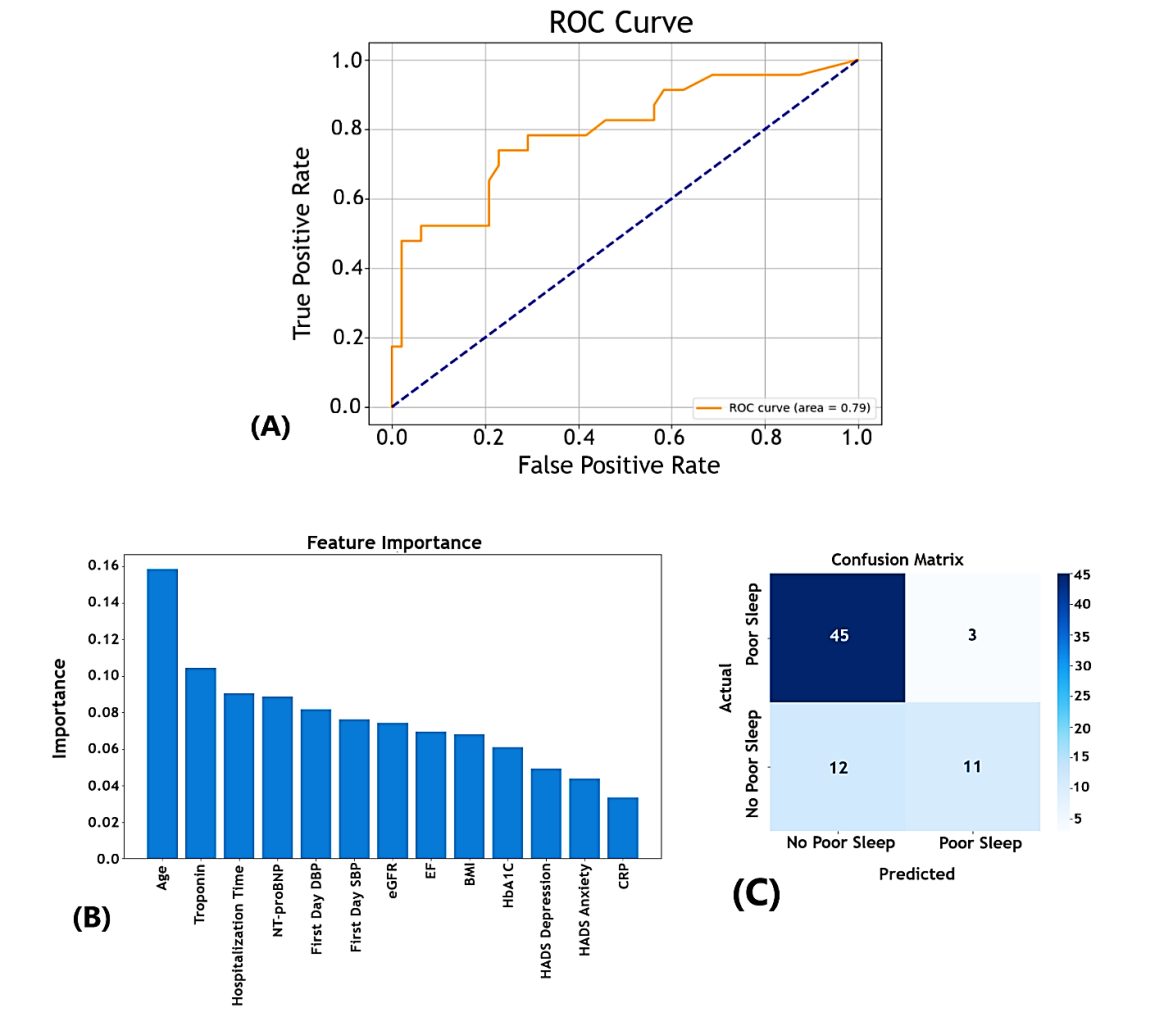
Diagnostic performance and key predictors of the random forest model for sleep quality in hospitalized heart failure patients

 The RF model’s ability to predict poor sleep quality was evaluated using an ROC curve, yielding an AUC of 0.79 (Figure 1A). This indicates reasonable discrimination between patients with good and poor sleep quality, with the model performing better than random guessing. The feature importance analysis from our model highlights age as the most critical predictor, followed by troponin and hospitalization time. Other significant factors included NT-proBNP, first-day DBP, and eGFR. Psychosocial factors such as depression and state anxiety, along with CRP, exhibited lower importance, suggesting a lesser influence on the model’s predictions (Figure 1B).

###  Classification metrics

- *Confusion Matrix:* The model correctly predicted 45 cases of good sleep quality (true negatives) and 11 cases of poor sleep quality (true positives) but misclassified 12 false negatives and 3 false positives (Figure 1C).
- *Precision and Recall:* Good Sleep Quality (PSQI-Binary = 0): High precision (0.79) and recall (0.94) demonstrate strong performance in identifying patients with good sleep quality.
- *Good Sleep Quality (PSQI-Binary = 0):* High precision (0.79) and recall (0.94) demonstrated strong performance in identifying patients with good sleep quality.
- *Poor Sleep Quality (PSQI-Binary = 1):* While precision remained at 0.79, the recall was lower at 0.48, indicating that the model could successfully identify some patients with poor sleep quality but missed a significant portion.
- *Overall Accuracy:* The model achieved an accuracy of 79% with a macro-averaged F1-score of 0.73, reflecting a moderate balance between precision and recall across both classes.

###  Ordinary least squares regression analysis 

**Figure 2:**
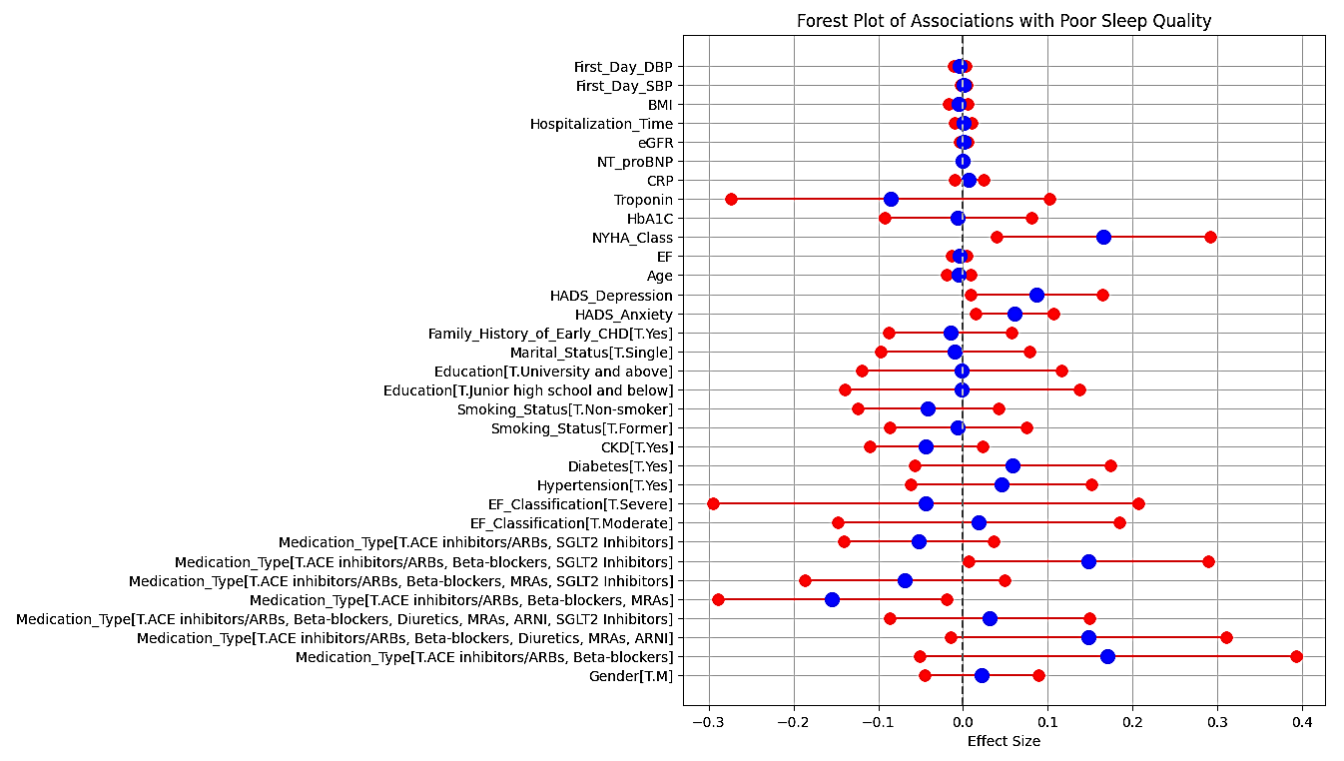
Forest plot of associations with poor sleep quality

Figure 2 (the corresponding OLS regression forest plot) illustrates the associations between clinical, demographic, and treatment variables with poor sleep quality in our cross-sectional study on 354 patients. The model’s R-squared value of 0.280 indicates that 28% of the variability in poor sleep quality can be explained, suggesting a moderate fit.

 The regression results are provided as follows:

- *Medication type:* “ACE inhibitors/ARBs, beta-blockers, and SGLT2 inhibitors” showed a positive association with poor sleep quality (coef = 0.1481, *P* = 0.040). Conversely, “ACE inhibitors/ARBs, beta-blockers, and MRAs” had a significant negative association (coef = -0.1547, *P* = 0.025).
- *Psychological factors:* Both “HADS-anxiety” and “HADS-depression” scores were significantly associated with poor sleep quality (*P* = 0.010 and 0.029, respectively).
- *NYHA class:* Higher NYHA class was positively associated with poor sleep quality (coef = 0.1655, *P* = 0.010).
- *Demographic and clinical variables:* Gender, age, smoking status, and comorbidities such as diabetes, HTN, and CKD revealed non-significant associations with poor sleep quality.

This forest plot in Figure 2 displays the effect sizes of various clinical, demographic, psychosocial, and treatment-related factors on the likelihood of poor sleep quality in patients with HF. The horizontal axis represents the effect size, with a vertical line at zero indicating no effect. Red and blue markers depict the effect sizes and their corresponding confidence intervals for each factor.

###  Partial dependence plots for predictors of poor sleep quality

**Figure 3:**
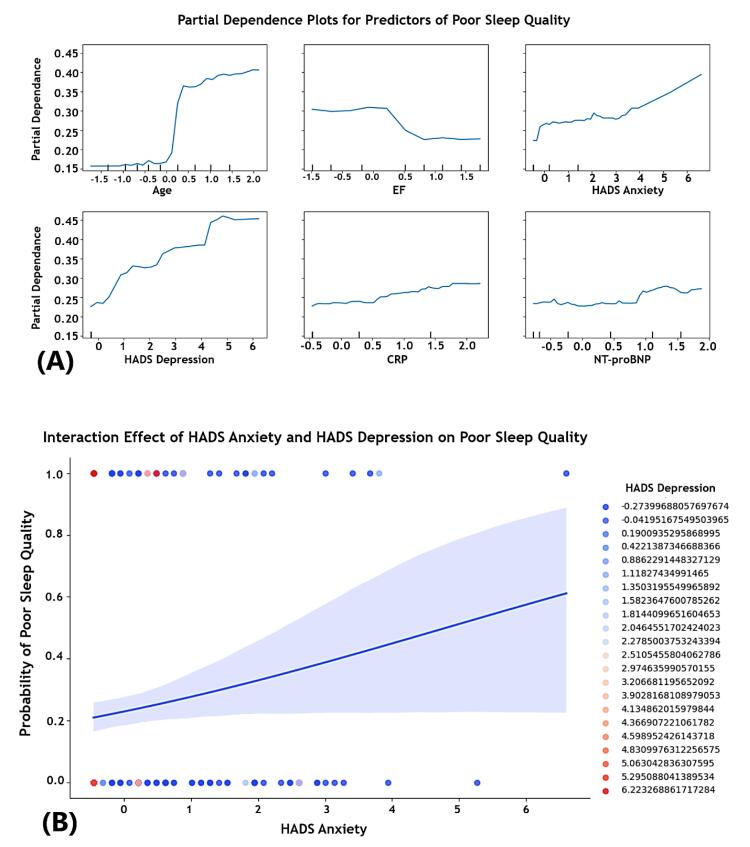
Partial dependence and interaction effects on poor sleep quality in heart failure patients

 PDPs were used to visualize the marginal effect of predictor variables on the probability of poor sleep quality, holding other variables constant (Figure 3A-3B).

- *Age:* A sharp increase was found in the probability of poor sleep quality with increasing age, implying that older age is associated with a higher likelihood of poor sleep, possibly due to comorbidities and changes in sleep patterns.
- *Ejection fraction:* A decreasing trend in poor sleep probability was observed as EF increases, stabilizing at higher EF levels. This suggests that patients with lower EF, indicative of HF, are at a higher risk of poor sleep quality.
- *Hospital anxiety and depression scale-anxiety and hospital anxiety and depression scale-depression:* Both anxiety and depression scores demonstrated a positive association with poor sleep quality. As these scores increased, the probability of poor sleep also represented a significant increase.
- *C-reactive protein:* The PDP for CRP indicated a modest upward trend, confirming that higher levels of CRP, a marker of inflammation, may be linked to an increased likelihood of poor sleep.
- *N-terminal prohormone of brain natriuretic peptide:* The plot for NT-proBNP remained relatively flat with slight fluctuations, suggesting a minimal impact on the probability of poor sleep quality.

###  Multivariable logistic regression analysis 

 In this multivariable logistic regression analysis, age consistently emerged as a significant predictor of poor sleep quality (Table 2). Each additional year of age was associated with an increased likelihood of experiencing poor sleep quality (odds ratio [OR] = 1.19, *P* < 0.001). Gender, however, had no statistically significant effect on sleep quality.

**Table 2:** Logistic regression analysis of the association between psychological symptoms and sleep quality

**Variables**	**Model 1**		**Model 2**		**Model 3**		**Model 4**		**Model 5**	
	**OR (95%CI)**	***P*** ** value**	**OR (95% CI)**	***P*** ** value**	**OR (95% CI)**	***P*** ** value**	**OR (95% CI)**	***P*** ** value**	**OR (95% CI)**	***P*** ** value**
Age	1.19 (1.13–1.25)	0.001	1.18 (1.11–1.26)	0.001	1.16 (1.06–1.27)	0.001	1.16 (1.06–1.27)	0.001	1.16 (1.06–1.26)	0.001
Gender	1.15 (0.59–2.24)	0.673	1.14 (0.58–2.21)	0.705	1.13 (0.57–2.23)	0.723	1.02 (0.51–2.06)	0.945	1.36 (0.66–2.77)	0.399
Overweight/obesity			1.24 (0.49–3.12)	0.649	1.41 (0.55–3.63)	0.477	1.69 (0.62–4.56)	0.303	1.30 (0.49–3.43)	0.593
Education			0.91 (0.50–1.68)	0.773	0.93 (0.50–1.73)	0.818	0.89 (0.47–1.67)	0.713	0.97 (0.51–1.84)	0.925
Marital status			1.07 (0.41–2.76)	0.886	1.05 (0.40–2.79)	0.914	0.97 (0.36–2.63)	0.950	0.98 (0.36–2.70)	0.967
Hospitalization time			1.00 (0.93–1.07)	0.993	0.99 (0.90–1.08)	0.760	0.98 (0.90–1.08)	0.710	0.98 (0.89–1.07)	0.590
Family history of early CHD			1.16 (0.58–2.31)	0.676	1.15 (0.56–2.35)	0.698	0.97 (0.46–2.05)	0.939	1.19 (0.57–2.46)	0.641
Smoking status					0.77 (0.50–1.19)	0.244	0.74 (0.45–1.21)	0.181	0.73 (0.47–1.13)	0.160
Hypertension					1.27 (0.63–2.56)	0.500	1.23 (0.60–2.49)	0.571	1.18 (0.58–2.43)	0.643
Diabetes					1.52 (0.75–3.09)	0.248	1.57 (0.76–3.23)	0.222	1.53 (0.74–3.17)	0.251
CKD					0.57 (0.29–1.13)	0.105	0.62 (0.31–1.25)	0.184	0.54 (0.27–1.10)	0.089
CRP					1.08 (0.94–1.24)	0.302	1.08 (0.94–1.25)	0.261	1.07 (0.93–1.23)	0.348
EF					0.99 (0.94–1.04)	0.686	0.99 (0.94–1.04)	0.673	0.98 (0.93–1.03)	0.424
HADS depression							2.08 (1.07–4.05)	0.031		
HADS anxiety									1.89 (1.28–2.79)	0.001

*Note*. OR: Odds ratio; CI: Confidence interval; CHD: Coronary heart disease; HADS: Hospital Anxiety and Depression Scale; BMI: Body mass index; CKD: Chronic kidney disease; EF: Ejection fraction; CRP: C-reactive protein **Model 1**: Adjusted for age and gender. **Model 2**: Model 1 + overweight or obesity (BMI ≥ 24 kg/m^2^), education, marital status, hospitalization time, and family history of early-onset coronary heart disease. **Model 3**: Model 2 + smoking status, hypertension, diabetes, CKD, CRP, and left ventricular EF. **Model 4**: Model 3 + HADS-depression. **Model 5**: Model 3 + HADS-anxiety.

 When sociodemographic variables such as obesity, education, and marital status were introduced in Model 2, age remained a significant predictor of poor sleep quality (OR = 1.18, *P* < 0.001). Despite the addition of these factors, none of them were statistically significant and could not noticeably enhance the model’s predictive power.

 Model 3 incorporated comorbidities, including smoking, HTN, and diabetes. Age continued to be a significant predictor (OR = 1.16, *P* = 0.001), while none of the added comorbidities reached statistical significance. The model revealed moderate improvement in fit, whereas the clinical factors did not provide a significant predictive value.

 With the inclusion of depression as a variable in Model 4, the analysis confirmed that age (OR = 1.16, *P* = 0.001) and depression (OR = 2.08, *P* = 0.031) were significant predictors of poor sleep quality. The presence of depression markedly increased the likelihood of experiencing poor sleep, highlighting the role of psychological factors.

 Model 5 further expanded the analysis by adding anxiety as a variable. Both age (OR = 1.16, *P* = 0.001) and anxiety (OR = 1.89, *P* = 0.001) were found to be significant predictors of poor sleep quality. This model underscores the impact of anxiety on sleep disturbances, demonstrating that psychological factors, particularly anxiety and depression, are critical in understanding poor sleep quality in this population.

## Discussion

 This descriptive-analytical, cross-sectional study involved 354 hospitalized HF patients over one year in Chamran Heart Center, Isfahan, Iran (September 2023-2024). Sleep quality, anxiety, and depression were assessed using the PSQI and HADS, respectively. Statistical analysis revealed that poor sleep quality is multifactorial, with significant predictors including advanced age, reduced cardiac function, higher BP, and complex medication regimens. Additionally, the strong association between psychological factors (e.g., anxiety and depression) and poor sleep highlights the need for a multidisciplinary approach to managing HF patients.

 Some studies evaluated sleep quality disturbance among patients with different CVDs and its association with psychological outcomes and multiple variables. For instance, Matsuda et al^12^ found that 43% of patients hospitalized with CVDs had poor sleep quality, which was independently associated with higher depression and anxiety scores. Notably, women showed a stronger association between poor sleep quality and depression compared to men.^12^ Similarly, Cheng et al reported that poor sleep quality was linked to anxiety and depression in Chinese patients with CHD, with anxiety emerging as the primary driver of poor sleep in cases where both conditions were present.^26^

 Among CVDs, HF, in particular, presents a complex interplay between sleep quality and psychological health. Aria et al^17^ concluded that 80% of HF patients experience significant sleep disturbances, which are strongly correlated with anxiety and depression. The total PSQI score in these patients is significantly correlated with anxiety (r = 0.216, *P* = 0.035) and depression (r = 0.351, *P* = 0.000), emphasizing the close relationship between sleep quality and mental health in this population. This is in line with the results of our study, indicating that patients with poor sleep quality had significantly higher depression (HADS-depression: 5.36 ± 0.70 vs. 5.10 ± 0.40, *P* < 0.001) and anxiety (HADS-anxiety: 4.84 ± 1.32 vs. 4.30 ± 0.68, *P* < 0.001) scores compared to those with good sleep quality (HADS-depression: 5.10 ± 0.40 and HADS-anxiety: 4.30 ± 0.68).

 Several predictors and variables are consistently associated with PSQI in patients with CVDs, particularly HF. These predictors can be categorized into demographic, clinical, psychological, and socioeconomic factors. Matsuda et al^12^ found that age and gender are significant predictors, as older age is strongly correlated with poor sleep quality, while women demonstrated a stronger association between poor sleep quality and depressive symptoms compared to men. Our observation showed that poor sleep quality was significantly more frequent in older-aged patients (70.00 ± 6.30 years vs. 62.58 ± 7.39 years, *P* < 0.001), while gender could not significantly impact sleep quality (*P* = 0.659). Additionally, Kohanmoo et al^27^ concluded that lower educational levels and income are associated with poorer sleep quality, particularly among female patients. Nonetheless, our results confirmed that sociodemographic variables such as obesity, education, and marital status did not significantly affect poor sleep quality.

 Multiple clinical factors are predictive of poor sleep quality in cardiovascular patients. For instance, Jun et al^28^ revealed that left ventricular hypertrophy is an independent predictor of poor sleep quality in patients with CKD, which may contribute to cardiovascular damage. However, Edalat-Nejad et al^29^ reported that in hemodialysis patients, diabetic nephropathy and body pain were significant predictors of poor sleep quality.

 Our results contradict those of Kohanmoo et al,^27^ indicating that socioeconomic status, particularly lower education and income levels, significantly affected sleep quality, especially among women with CAD. In our study, when sociodemographic variables such as obesity, education, and marital status were introduced in a multivariable logistic regression analysis (Model 2), age remained a significant predictor of poor sleep quality (OR = 1.18, *P* < 0.001), but none of the sociodemographic factors reached statistical significance, nor did they enhance the model’s predictive power.

 The presence of comorbidities, such as diabetes and HTN, also contributes to poor sleep quality. Our analysis confirmed that diabetes prevalence did not differ significantly between patients with poor quality of sleep (58.33%), while DBP was higher in this group. Furthermore, some studies demonstrated the use of certain medications has been associated with worse sleep outcomes in cardiovascular patients. Lin et al^30^ found that diuretic treatment could reduce sleep quality in patients with obstructive sleep apnea and cardiovascular comorbidities. However, Nerbass et al^31^ indicated that calcium channel blockers, often used in conjunction with diuretics, were linked to shorter sleep durations in hypertensive patients with sleep apnea, further aggravating sleep issues. Our regression analysis showed that “ACE inhibitors/ARBs, beta-blockers, and SGLT2 inhibitors” were positively associated with poor sleep quality (coef = 0.1481, *P* = 0.040), while “ACE inhibitors/ARBs, beta-blockers, and MRAs” had a negative association (coef = -0.1547, *P* = 0.025).

 Predictive models and special statistical methods were employed to assess the relationship between poor sleep quality and cardiovascular outcomes, including clinical and subclinical conditions. These models integrate multiple predictors, ranging from demographic to clinical factors, and use advanced techniques to refine predictions.

 Among the models used in this study, the RF model represented the best classification performance, achieving an AUC of 0.79 on the ROC curve. This performance indicates a moderate ability to differentiate between patients with and without poor sleep quality, making it the most effective model for classification tasks. The RF model also identified key predictors, including age, troponin levels, and hospitalization duration, through its feature importance analysis. In contrast, the predictive model developed by Park et al^32^ using an SVM achieved a much higher precision (87.5%) in predicting cardiovascular events, focusing on sleep-disordered breathing and incorporating ECG features and clinical risk factors. This suggests that SVM, optimized with recursive feature elimination, may be better suited for identifying specific cardiovascular outcomes.

 Similarly, Guo et al^33^ utilized hierarchical regression to explore predictors of sleep quality in hospitalized cardiovascular patients, identifying that age and depression significantly affected sleep outcomes. Their findings emphasized the impact of psychological and sociodemographic factors, which aligns with our observations but with different model implications for clinical application.

 Likewise, our multivariable logistic regression analysis revealed that age consistently emerged as a significant predictor of poor sleep quality across all models (OR = 1.19, *P* < 0.001 in Model 1), with psychological factors such as depression (OR = 2.08, *P* = 0.031) and anxiety (OR = 1.89, *P* = 0.001) also being strong predictors in Model 4 and 5, respectively. Gender, sociodemographic variables, and clinical comorbidities had no significant effect on sleep quality.

 In comparison, Cheng et al^26^ also used multivariable logistic regression to examine the association between psychological factors and sleep quality. Their analysis confirmed that anxiety symptoms were robust predictors of poor sleep quality across all models (OR = 1.12–1.14, *P* < 0.001), which conforms to our findings. Depression symptoms were also significant predictors in their initial models (OR = 1.11, *P* = 0.001), but unlike our results, the association between poor sleep quality and depression became non-significant after adjusting for anxiety symptoms (*P* = 0.491) in their final model.

 While both studies highlight the critical role of psychological factors in predicting poor sleep quality, Cheng et al^26^ observed a shift in the significance of depression once anxiety was accounted for, suggesting a more complex interaction between these psychological factors. In contrast, our analysis demonstrated that both anxiety and depression remained significant predictors even when both were included in the model, underscoring their independent contributions to sleep disturbances in our population. Additionally, our study emphasized the consistent influence of age on sleep quality, which was not as prominently addressed in the models of Cheng et al.

 Our cross-sectional study on hospitalized HF patients presents valuable insights but has notable limitations. The cross-sectional design restricts causal inferences between sleep disturbances and psychological distress, indicating the need for longitudinal studies to establish temporal relationships. Considering that the study was conducted exclusively at one center, the findings may not be generalizable to broader populations. Moreover, the reliance on self-reported data (PSQI and HADS) introduces potential bias, as subjective perceptions may not fully align with objective measures. Additionally, excluding patients with severe neuropsychiatric conditions may underestimate the overall burden of sleep disturbances in this population. Finally, the effects of various medications were not accounted for, and although the sample size of 354 patients was substantial, larger studies might be needed to detect more subtle associations.

 On the other hand, the study’s strengths include rigorous multivariable analysis, the use of validated tools, and a focus on a vulnerable, high-risk population. Incorporating predictive modeling with RF algorithms adds clinical relevance by offering insights for targeted interventions. By integrating psychosocial and clinical variables, this study could provide a comprehensive understanding of factors influencing sleep quality in HF patients, contributing valuable knowledge for clinical practice and future research.

**Highlights**

- Advanced age and lower EF were strong predictors of poor sleep in heart failure (HF) patients.
- Poor sleep was linked to higher SBP, complex meds, and high depression/anxiety scores.
- Angiotensin-converting enzyme (ACE) inhibitors, beta-blockers, and diuretics worsened sleep quality in HF patients.
- The random forest (RF) model showed an area under the curve (AUC) of 0.79, with age as the top predictor of poor sleep.
- Troponin levels and hospitalization time also contributed to poor sleep predictions.

## Conclusion

 This study highlights the multifactorial nature of poor sleep quality in hospitalized HF patients, with advanced age, reduced EF, and complex medication regimens as key clinical predictors. In addition, elevated anxiety and depression underscore the psychosocial dimensions of these disturbances. Our findings also stress the need for integrated management combining pharmacological optimization with psychosocial interventions to improve outcomes. Eventually, the predictive utility of the RF model provides a basis for refining personalized care and reducing the healthcare burden of sleep disturbances in HF.

## Competing Interests

 All authors report that they have no potential conflict of interests.

## Ethical Approval

 The co-authors guarantee the legitimacy and soundness of this study and ensure compliance with all the principles of the Declaration of Helsinki. The ethics committee of Isfahan University of Medical Sciences approved the study (ethical code IR.ARI.MUI.REC.1402.216 and research code 2402213). All patients participating in the study provided informed consent with strict confidentiality prior to completing the required data collection forms throughout the research process.

## Funding

 Not applicable.
